# The genome sequence of the peppered moth, *Biston betularia *Linnaeus, 1758

**DOI:** 10.12688/wellcomeopenres.17578.1

**Published:** 2022-03-17

**Authors:** Douglas Boyes, Charlotte Wright, University of Oxford and Wytham Woods Genome Acquisition Lab, Darwin Tree of Life Barcoding collective, Wellcome Sanger Institute Tree of Life programme, Wellcome Sanger Institute Scientific Operations: DNA Pipelines collective, Tree of Life Core Informatics collective, Darwin Tree of Life Consortium

**Affiliations:** 1 UK Centre for Ecology and Hydrology, Wallingford, Oxfordshire, UK; 2 Tree of Life, Wellcome Sanger Institute, Cambridge, UK

**Keywords:** Biston betularia, peppered moth, genome sequence, chromosomal, Lepidoptera

## Abstract

We present a genome assembly from an individual male *Biston betularia *(the peppered moth; Arthropoda; Insecta; Lepidoptera; Geometridae). The genome sequence is 405 megabases in span. The majority of the assembly (99.99%) is scaffolded into 31 chromosomal pseudomolecules, with the Z sex chromosome assembled.Gene annotation of this assembly on Ensembl has identified 12,251 protein coding genes.

## Species taxonomy

Eukaryota; Metazoa; Ecdysozoa; Arthropoda; Hexapoda; Insecta; Pterygota; Neoptera; Endopterygota; Lepidoptera; Glossata; Ditrysia; Geometroidea; Geometridae; Ennominae; Biston; *Biston betularia* Linnaeus, 1758 (NCBI:txid82595).

## Background

The peppered moth, *Biston betularia*, is widely distributed throughout Europe, Asia and North America. The species has one generation per year, with adults flying between May and August in England. Larvae mimic twigs in their form, and can even change colour to match their surroundings ( Eacock *et al.*, 2019; Eacock *et al.*, 2017). Larvae feed on a wide variety of deciduous trees and bushes, including birch, blackthorn and roses. Individuals overwinter underground as pupae. A pale *typica* form is white, peppered with black on wings and body while a melanic, *carbonaria* form with white spots is associated with areas with higher atmospheric pollution levels. The two forms can interbreed resulting in intermediate forms. Genetically distinct are insularia, with a range of intermediate colour patterns. Industrial melanism in the peppered moth is a classic example of rapid adaptive response to environmental change Cook, 2003). High levels of coal pollution during the industrial revolution led to a rise in the frequency of the *carbonaria* form in urban areas due to selective predation. In recent decades, the frequency of the melanic form has decreased, in line with reduced pollution levels. The genetic basis of industrial melanism has been attributed to the insertion of a large transposable element into the first intron of the gene *cortex* ( Van’t Hof *et al.*, 2016). This event occurred in Britain in approximately 1819, in line with the historical record ( Van’t Hof *et al.*, 2016). Interestingly, *cortex* has been repeatedly associated with colour pattern variation in diverse lepidopteran species, including in *Heliconius* butterflies where it is a major determinant of scale cell identity ( Livraghi *et al.*, 2021; Nadeau *et al.*, 2016; Van Belleghem *et al.*, 2017). *Biston betularia* has a karyotype of 31 chromosomes ( Van’t Hof *et al.*, 2013).

## Genome sequence report

**Figure 1.**
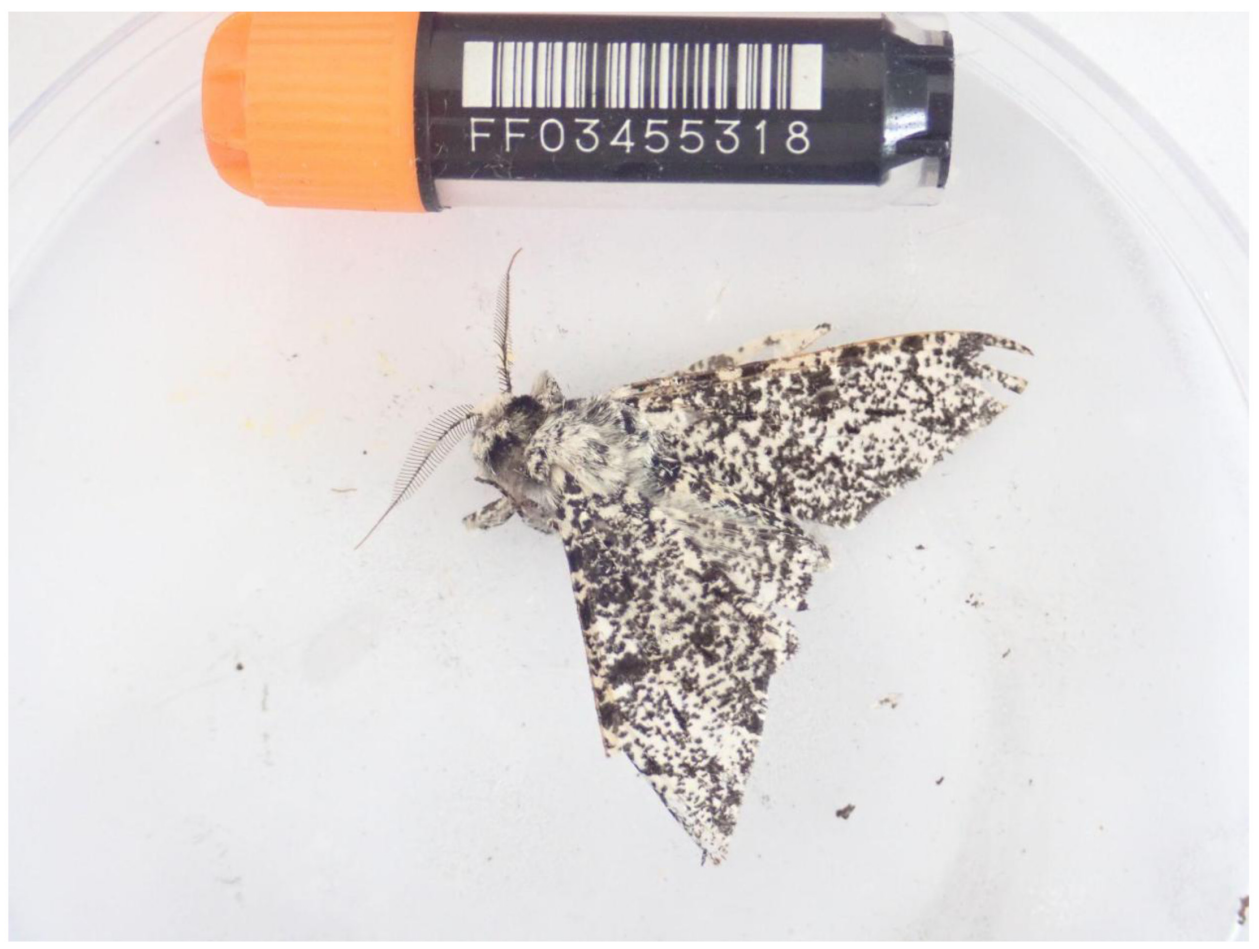
Image of the ilBisBetu1 specimen taken prior to preservation and processing. Specimen shown next to FluidX storage tube, 43.9 mm in length.

The genome was sequenced from one male *B. betularia* ( Figure 1) collected from Wytham Woods, Oxfordshire (biological vice-county: Berkshire), UK (latitude 51.772, longitude -1.338). A total of 27-fold coverage in Pacific Biosciences single-molecule long reads and 91-fold coverage in 10X Genomics read clouds were generated. Primary assembly contigs were scaffolded with chromosome conformation Hi-C data. Manual assembly curation corrected 8 missing/misjoins, reducing the scaffold number by 15.79% and increasing the scaffold N50 by 2.39%.

**Figure 2.**
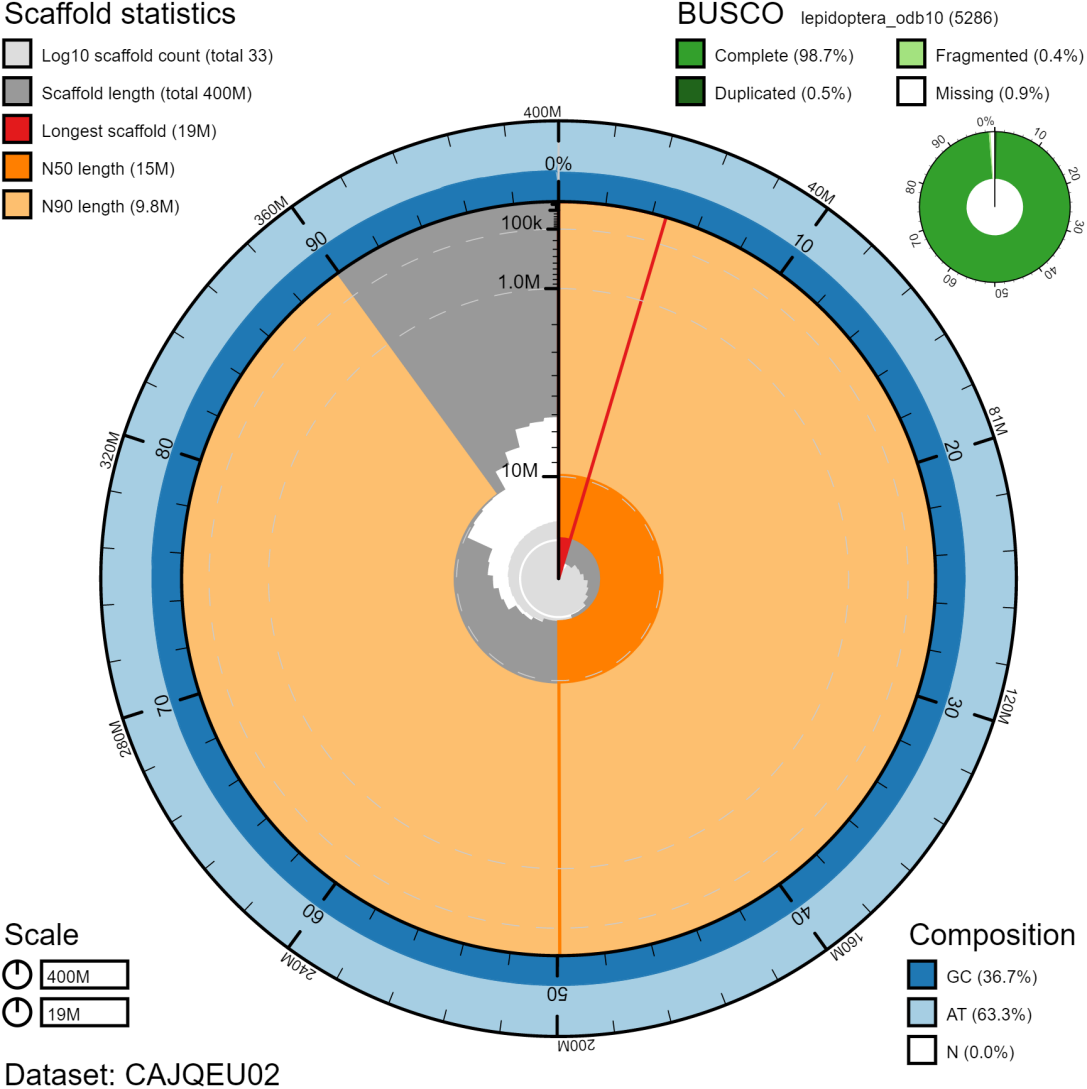
Genome assembly of *Biston betularia*, ilBisBetu1.1: metrics. The BlobToolKit Snailplot shows N50 metrics and BUSCO gene completeness. The main plot is divided into 1,000 size-ordered bins around the circumference with each bin representing 0.1% of the 404,525,905 bp assembly. The distribution of scaffold lengths is shown in dark grey with the plot radius scaled to the longest scaffold present in the assembly (18,795,478 bp, shown in red). Orange and pale-orange arcs show the N50 and N90 scaffold lengths (14,733,994 and 9,789,996 bp), respectively. The pale grey spiral shows the cumulative scaffold count on a log scale with white scale lines showing successive orders of magnitude. The blue and pale-blue area around the outside of the plot shows the distribution of GC, AT and N percentages in the same bins as the inner plot. A summary of complete, fragmented, duplicated and missing BUSCO genes in the lepidoptera_odb10 set is shown in the top right. An interactive version of this figure is available at https://blobtoolkit.genomehubs.org/view/ilBisBetu1.2/dataset/CAJQEU02/snail.

The final assembly has a total length of 405 Mb in 32 sequence scaffolds with a scaffold N50 of 14.7 Mb ( Table 1). The majority of the assembly sequence (99.99%) was assigned to 31 chromosomal-level scaffolds, representing 30 autosomes (numbered by sequence length, and the Z chromosome ( Figure 2– Figure 5; Table 2). The assembly has a BUSCO v5.1.2 ( Manni *et al.*, 2021) completeness of 98.7% (single 98.3%, duplicated 0.4%) using the lepidoptera_odb10 reference set. While not fully phased, the assembly deposited is of one haplotype. Contigs corresponding to the second haplotype have also been deposited.

**Table 1.** Genome data for *Biston betularia*, ilBisBetu1.1.

*Project accession data*	
Assembly identifier	ilBisBetu1.2
Species	*Biston betularia*
Specimen	ilBisBetu1
NCBI taxonomy ID	NCBI:txid82595
BioProject	PRJEB43794
BioSample ID	SAMEA7520512
Isolate information	Male, thorax/abdomen (genome assembly), head (Hi-C)
*Raw data accessions*	
PacificBiosciences SEQUEL II	ERR6412032, ERR6412367, ERR6436365
10X Genomics Illumina	ERR6054592, ERR6054595
Hi-C Illumina	ERR6054591
*Genome assembly*	
Assembly accession	GCA_905404145.2
Accession of alternate haplotype	GCA_905404215.1
Span (Mb)	405
Number of contigs	43
Contig N50 length (Mb)	14.0
Number of scaffolds	32
Scaffold N50 length (Mb)	14.7
Longest scaffold (Mb)	17.1
BUSCO * genome score	C:98.7%[S:98.3%,D:0.4%],F:0.4%,M:0.9%,n:5286
*Genome annotation*	
Number of protein-coding genes	12,251
Average coding sequence length (bp)	1,547.96
Average number of exons per transcript	7.88
Average exon length (bp)	329.93
Average intron size (bp)	2,082.45

*BUSCO scores based on the lepidoptera_odb10 BUSCO set using v5.2.2. C= complete [S= single copy, D=duplicated], F=fragmented, M=missing, n=number of orthologues in comparison. A full set of BUSCO scores is available at https://blobtoolkit.genomehubs.org/view/ilBisBetu1.1/dataset/CAJQEU01/busco.

**Figure 3.**
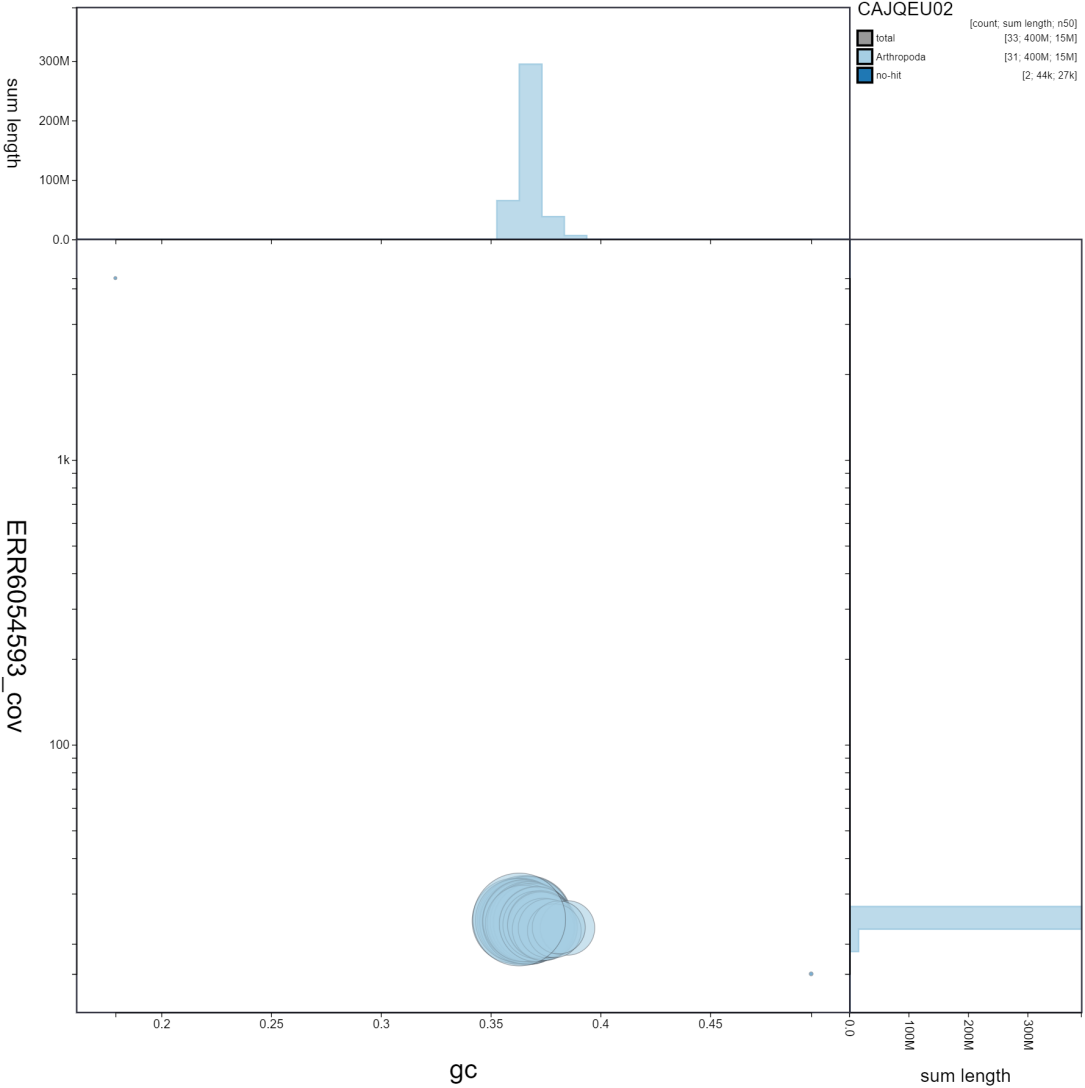
Genome assembly of *Biston betularia*, ilBisBetu1.2: GC coverage. BlobToolKit GC-coverage plot. Scaffolds are coloured by phylum. Circles are sized in proportion to scaffold length Histograms show the distribution of scaffold length sum along each axis. An interactive version of this figure is available at https://blobtoolkit.genomehubs.org/view/ilBisBetu1.2/dataset/CAJQEU02/blob.

**Figure 4.**
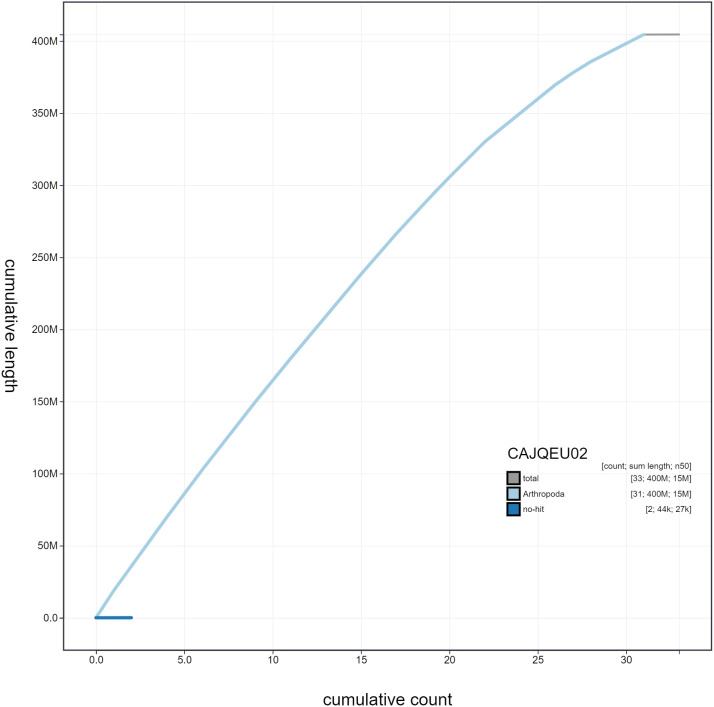
Genome assembly of *Biston betularia*, ilBisBetu1.2: cumulative sequence. BlobToolKit cumulative sequence plot. The grey line shows cumulative length for all scaffolds. Coloured lines show cumulative lengths of scaffolds assigned to each phylum using the buscogenes taxrule. An interactive version of this figure is available at https://blobtoolkit.genomehubs.org/view/ilBisBetu1.2/dataset/CAJQEU02/cumulative.

**Figure 5.**
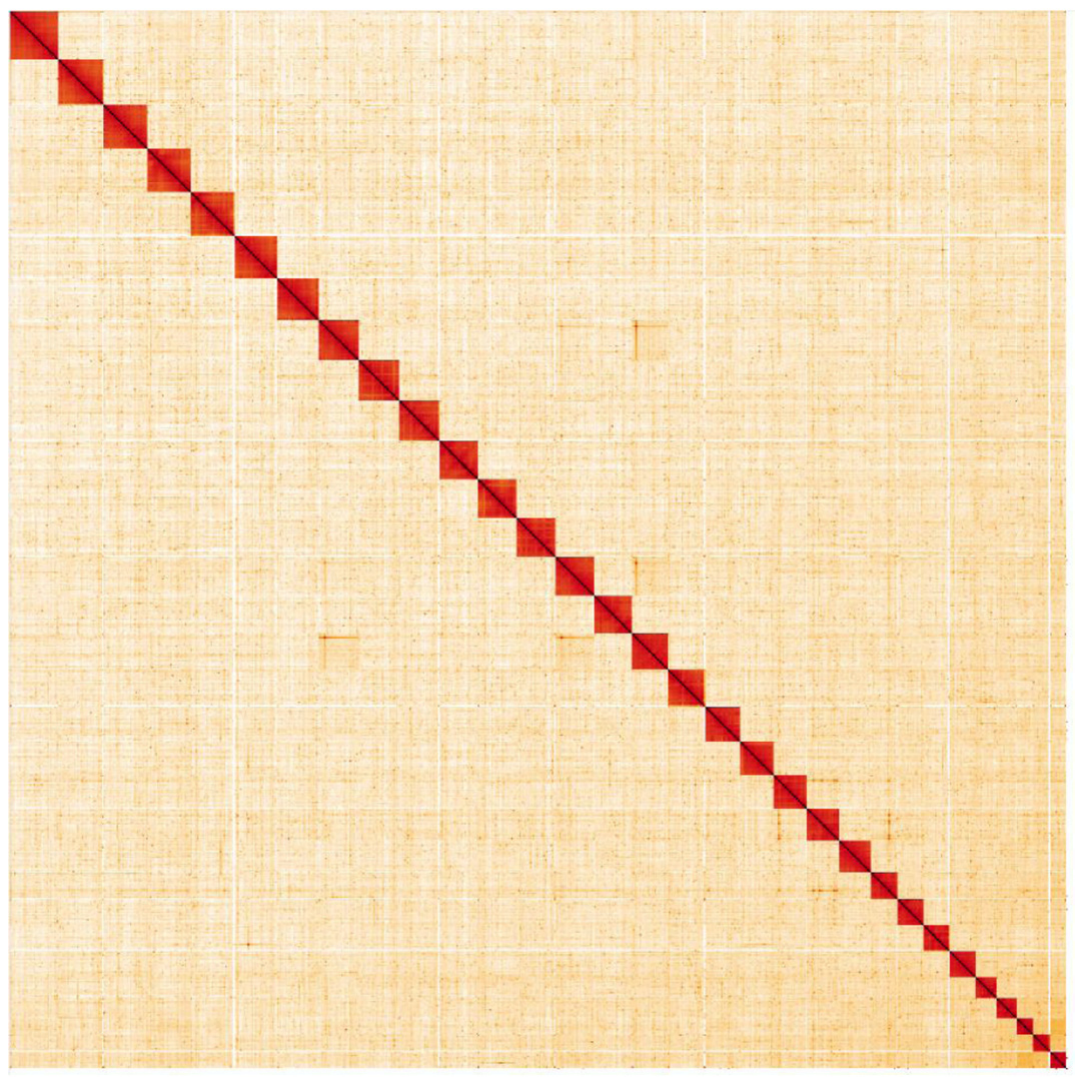
Genome assembly of *Biston betularia*, ilBisBetu1.2: Hi-C contact map. Hi-C contact map of the ilBisBetu1.2 assembly, visualised in HiGlass. Chromosomes are shown in order of size from left to right and top to bottom. An interactive version of this map is available here.

**Table 2.** Chromosomal pseudomolecules in the genome assembly of *Biston betularia*, ilBisBetu1.1.

INSDC accession	Chromosome	Size (Mb)	GC%
FR989863.1	1	17.11	36.7
FR989864.1	2	17.08	36.5
FR989865.1	3	16.63	36.7
FR989866.1	4	16.55	36.7
FR989867.1	5	16.27	36.4
FR989868.1	6	15.89	36.1
FR989869.1	7	15.72	36.4
FR989870.1	8	15.42	36.3
FR989871.1	9	15.10	36.4
FR989872.1	10	15.08	36.2
FR989873.1	11	14.79	36.5
FR989874.1	12	14.73	36.7
FR989875.1	13	14.73	36.5
FR989876.1	14	14.39	36.5
FR989877.1	15	14.09	36.7
FR989878.1	16	14.01	36.8
FR989879.1	17	13.31	36.9
FR989880.1	18	13.14	36.6
FR989881.1	19	12.73	36.5
FR989882.1	20	12.30	37.1
FR989883.1	21	12.17	37.1
FR989884.1	22	10.08	37.4
FR989885.1	23	9.97	37.1
FR989886.1	24	9.84	37.3
FR989887.1	25	9.79	37.3
FR989888.1	26	8.39	37.4
FR989889.1	27	7.55	37.6
FR989890.1	28	6.44	38.5
FR989891.1	29	6.28	37.9
FR989892.1	30	6.08	38.1
FR989862.1	Z	18.80	36.3
FR989893.2	MT	0.02	18
-	Unplaced	0.03	49.6

## Genome annotation report

The ilBisBetu1.1 genome was annotated using the Ensembl rapid annotation pipeline ( Table 1; https://rapid.ensembl.org/Biston_betularia_GCA_905404145.1/). The resulting annotation includes 19,758 transcribed mRNAs from 12,251 protein-coding and 2.985 non-coding genes. There are 1.61 coding transcripts per gene and 7.88 exons per transcript.

## Methods

### Sample acquisition and DNA extraction

A single male *B. betularia* (ilBisBetu1) was collected from Wytham Woods, Oxfordshire (biological vice-county: Berkshire), UK (latitude 51.772, longitude -1.338) by Douglas Boyes, UKCEH, using a light trap. The sample was identified by the same individual, and preserved on dry ice.

DNA was extracted at the Tree of Life laboratory, Wellcome Sanger Institute. The ilBisBetu1 sample was weighed and dissected on dry ice with tissue set aside for Hi-C sequencing. Thorax tissue was cryogenically disrupted to a fine powder using a Covaris cryoPREP Automated Dry Pulveriser, receiving multiple impacts. Fragment size analysis of 0.01–0.5 ng of DNA was then performed using an Agilent FemtoPulse. High molecular weight (HMW) DNA was extracted using the Qiagen MagAttract HMW DNA extraction kit. Low molecular weight DNA was removed from a 200-ng aliquot of extracted DNA using 0.8X AMpure XP purification kit prior to 10X Chromium sequencing; a minimum of 50 ng DNA was submitted for 10X sequencing. HMW DNA was sheared into an average fragment size between 12–20 kb in a Megaruptor 3 system with speed setting 30. Sheared DNA was purified by solid-phase reversible immobilisation using AMPure PB beads with a 1.8X ratio of beads to sample to remove the shorter fragments and concentrate the DNA sample. The concentration of the sheared and purified DNA was assessed using a Nanodrop spectrophotometer and Qubit Fluorometer and Qubit dsDNA High Sensitivity Assay kit. Fragment size distribution was evaluated by running the sample on the FemtoPulse system.

### Sequencing

Pacific Biosciences HiFi circular consensus and 10X Genomics Chromium read cloud sequencing libraries were constructed according to the manufacturers’ instructions. Sequencing was performed by the Scientific Operations core at the Wellcome Sanger Institute on Pacific Biosciences SEQUEL II (HiFi) and Illumina HiSeq X (10X) instruments. Hi-C data were generated from head tissue using the Arima Hi-C+ kit and sequenced on HiSeq X.

### Genome assembly

Assembly was carried out with Hifiasm ( Cheng *et al.*, 2021); haplotypic duplication was identified and removed with purge_dups ( Guan *et al.*, 2020). One round of polishing was performed by aligning 10X Genomics read data to the assembly with longranger align, calling variants with freebayes ( Garrison & Marth, 2012). The assembly was then scaffolded with Hi-C data ( Rao *et al.*, 2014) using SALSA2 ( Ghurye *et al.*, 2019). The assembly was checked for contamination and corrected using the gEVAL system ( Chow *et al.*, 2016) as described previously ( Howe *et al.*, 2021). Manual curation ( Howe *et al.*, 2021) was performed using gEVAL, HiGlass ( Kerpedjiev *et al.*, 2018) and Pretext. The mitochondrial genome was assembled using MitoHiFi ( Uliano-Silva *et al.*, 2021), which performs annotation using MitoFinder ( Allio *et al.*, 2020). The genome was analysed and BUSCO scores generated within the BlobToolKit environment ( Challis *et al.*, 2020). Table 3 contains a list of all software tool versions used, where appropriate.

**Table 3.** Software tools used.

Software tool	Version	Source
Hifiasm	0.12	Cheng *et al.*, 2021
purge_dups	1.2.3	Guan *et al.*, 2020
SALSA2	2.2	Ghurye *et al.*, 2019
longranger align	2.2.2	https://support.10xgenomics. com/genome-exome/ software/pipelines/latest/ advanced/other-pipelines
freebayes	1.3.1-17- gaa2ace8	Garrison & Marth, 2012
MitoHiFi	1.0	Uliano-Silva *et al.*, 2021
gEVAL	N/A	Chow *et al.*, 2016
HiGlass	1.11.6	Kerpedjiev *et al.*, 2018
PretextView	0.1.x	https://github.com/wtsi-hpag/ PretextView
BlobToolKit	2.6.2	Challis *et al.*, 2020

### Genome annotation

The Ensembl gene annotation system ( Aken *et al.*, 2016) was used to generate annotation for the Biston betularia assembly ( GCA_905404145.1). Annotation was created primarily through alignment of transcriptomic data to the genome, with gap filling via protein to-genome alignments of a select set of proteins from UniProt ( UniProt Consortium, 2019).

### Ethics/compliance issues

The materials that have contributed to this genome note have been supplied by a Darwin Tree of Life Partner. The submission of materials by a Darwin Tree of Life Partner is subject to the Darwin Tree of Life Project Sampling Code of Practice. By agreeing with and signing up to the Sampling Code of Practice, the Darwin Tree of Life Partner agrees they will meet the legal and ethical requirements and standards set out within this document in respect of all samples acquired for, and supplied to, the Darwin Tree of Life Project. Each transfer of samples is further undertaken according to a Research Collaboration Agreement or Material Transfer Agreement entered into by the Darwin Tree of Life Partner, Genome Research Limited (operating as the Wellcome Sanger Institute), and in some circumstances other Darwin Tree of Life collaborators.

## Data availability

European Nucleotide Archive: Biston betularia (peppered moth). Accession number PRJEB43794; https://identifiers.org/ena.embl/PRJEB43794.

The genome sequence is released openly for reuse. The *B. betularia* genome sequencing initiative is part of the Darwin Tree of Life (DToL) project. All raw sequence data and the assembly have been deposited in INSDC databases. The genome will be annotated and presented through the Ensembl pipeline at the European Bioinformatics Institute. Raw data and assembly accession identifiers are reported in Table 1.

## Author information

Members of the University of Oxford and Wytham Woods Genome Acquisition Lab are listed here: https://doi.org/10.5281/zenodo.5746938.

Members of the Darwin Tree of Life Barcoding collective are listed here: https://doi.org/10.5281/zenodo.5744972.

Members of the Wellcome Sanger Institute Tree of Life programme are listed here: https://doi.org/10.5281/zenodo.6125027.

Members of Wellcome Sanger Institute Scientific Operations: DNA Pipelines collective are listed here: https://doi.org/10.5281/zenodo.5746904.

Members of the Tree of Life Core Informatics collective are listed here: https://doi.org/10.5281/zenodo.6125046.

Members of the Darwin Tree of Life Consortium are listed here: https://doi.org/10.5281/zenodo.5638618.
