# Supplementary material for: The genome sequence of the peppered moth, Biston betularia Linnaeus, 1758
Source: Wellcome Open Res. 2022 Mar 17;7:97. [Version 1] doi: 10.12688/wellcomeopenres.17578.1 (PMC10061035; doi:10.12688/wellcomeopenres.17578.1)
Supplement: Peer review history

# Peer review: Reviewer response for version 1

**DOI:** 10.21956/wellcomeopenres.19437.r55338

**Published:** 2023-03-29

**Authors:** Martina Dalíková

**Affiliations:** 1 The University of Kansas, Lawrence, Kansas, USA

The manuscript entitled “The genome sequence of the peppered moth, *Biston betularia* Linnaeus, 1758” presents the assembly of the genome of an iconic lepidopteran species. The text briefly describes the motivation, used methods and basic characterization of the assembly. The authors used recent sequencing and assembling methods which are reflected in the high quality of obtained results. The information provided is mostly sufficient for this type of manuscript, however, I would still appreciate it if the authors can clarify the following points:

1. What methods if any were used for genome size estimation? Or was this information obtained from previously published data? What are the comparison of estimated genome size and obtained assembly length?
2. Can you provide more information about the Ensemble annotation process? (e.g. what transcriptomes and protein sets were used).
3. Although the haploid peppered moth haploid chromosome number is 31, the authors obtained 32 scaffolds, the fate of the “extra” scaffold is unclear to me. Can authors provide more information on if this scaffold was merged with another scaffold during Hi-C data incorporation, did it remain an unassigned scaffold? If so can the assignment be done by comparing the sequence with the published linkage map?
4. The Hi-C map contains few higher density signals outside of the assigned chromosomes, can authors briefly comment on this?

Minor comment: missing “(“ before Cook, 2003 reference in the Background chapter

Are sufficient details of methods and materials provided to allow replication by others?

Partly

Is the rationale for creating the dataset(s) clearly described?

Yes

Are the datasets clearly presented in a useable and accessible format?

Partly

Are the protocols appropriate and is the work technically sound?

Yes

Reviewer Expertise:

Cytogenetics, genomics

I confirm that I have read this submission and believe that I have an appropriate level of expertise to confirm that it is of an acceptable scientific standard.

# Peer review: Reviewer response for version 1

**DOI:** 10.21956/wellcomeopenres.19437.r53325

**Published:** 2023-01-03

**Authors:** Martin Pippel

**Affiliations:** 1 Max Planck Institute of Molecular Cell Biology and Genetics, Dresden, Germany

“The genome sequence of the peppered moth, *Biston betularia*” from Boyes *et al. *describes a high-quality genome assembly based on PacBio HiFi reads, 10X Genomics read clouds and HiC data. 

 The data note is well structured and based on the described methods and the publicly available sequencing data also reproducible by the scientific community. The primary assembly has a very high contiguity already at the contig level.

 Methods:

 Genome assembly methods are a bit short and it could be hard to reproduce the assembly without further documentation of the used program arguments. Even if all tools were run in default mode, it would be worth mentioning. Some of the used tools, such as purge_dups depend itself on other programs like minimap2. But those could not be found in Table 3. I could not find any information if all variants from Freebayes were used for the error polishing or if a variant filtering step was applied beforehand.

 I do only have some minor comments and suggestions:

  

- I could not find the HiC read coverage in the Data Note.
- Additional statistics about the sequencing data (e.g. read length N50 of HiFi reads and 10X read clouds, kmer based genome size and heterozygosity estimates) and the final assembly (e.g. merqury QV scores, repeat content) would be nice to see as well.
- Error-polishing strategies of HiFi-based assemblies are currently under debate. I assume you applied bcftools consensus on the filtered Freebayes VCF files similar to the VGP assembly pipeline ( https://github.com/VGP/vgp-assembly/tree/master/pipeline). How big was the improvement based in the QV-score? Were the alternate contigs included in the longranger alignment step?
- It is great that you also included the ccs bam file, including the kinetics information. A further great feature would be to make the potential methylation sites (5mc in CpG islands) available for both assemblies.
- Why were different BUSCO versions used? Table 1: v5.2.2 versus Figure 2: v5.1.2.

Are sufficient details of methods and materials provided to allow replication by others?

Yes

Is the rationale for creating the dataset(s) clearly described?

Yes

Are the datasets clearly presented in a useable and accessible format?

Yes

Are the protocols appropriate and is the work technically sound?

Yes

Reviewer Expertise:

genome assembly and annotation

I confirm that I have read this submission and believe that I have an appropriate level of expertise to confirm that it is of an acceptable scientific standard.
